# Comparison of positioning errors and PTV expansion between the iSCOUT image-guidance system and onboard EPID in rectal cancer radiotherapy: a real-world study

**DOI:** 10.3389/fonc.2025.1671586

**Published:** 2026-01-12

**Authors:** Bao Wan, Lu Hou, Jiangtao Han, Yongtai Zheng, Chao Liu, Shuo Sun, Yu Zhao, Fukui Huan, Hongkai Wang, Tantan Li

**Affiliations:** Department of Radiation Oncology, National Cancer Center/National Clinical Research Center for Cancer/Cancer Hospital, Chinese Academy of Medical Sciences and Peking Union Medical College, Beijing, China

**Keywords:** image guidance, positioning error, PTV expansion, radiotherapy, rectal cancer

## Abstract

**Introduction:**

The iSCOUT orthogonal imaging system and onboard EPID system are commonly used for image-guidance positioning in conventional linear accelerators, but studies comparing the two are scarce. This research aims to analyze and compare the differences and consistency between the iSCOUT and onboard EPID systems in rectal cancer patients in the prone position.

**Methods:**

A retrospective analysis was conducted on 277 rectal cancer patients positioned prone on a Belly Board. During radiotherapy, at least three position verifications were performed in the first week, followed by weekly verifications. Both iSCOUT and onboard EPID image-guidance systems were used simultaneously for these verifications. Systematic and random errors, as well as the consistency of fixed positioning, were evaluated based on images from both systems taken in the same position. Patients were grouped by tumor location, BMI, age, and gender. The positioning errors and PTV (Planning Target Volume) margins for each group were calculated to analyze relevant patterns.

**Results:**

Kappa consistency in the leftright (LR) and superior-inferior (SI) directions was better compared to the anterior-posterior(AP) direction (LR = 0.794, SI = 0.852, AP = 0.610). Patients with tumors located in the upper rectal location require larger PTV expansions compared to those with middle or lower tumors. Obese patients with a BMI≥28 exhibited significantly larger PTV expansions than other groups. Age based grouping revealed an inverse relationship between age and PTV margin expansion. No significant differences were observed when grouped by gender.

**Discussion:**

The iSCOUT and EPID systems demonstrate good clinical agreement. Optimal PTV margins in rectal cancer radiotherapy should be individualized, with special consideration given to tumor location (upper rectum), high BMI, and advanced patient age.

## 1 Introduction

The Electronic Portal Imaging Device (EPID) is an essential auxiliary tool in modern radiotherapy, mounted on linear accelerators to capture treatment images at megavoltage (MV) levels (1, 2). EPID primarily works by utilizing X-rays to produce two-dimensional fluoroscopic images of the patient. These images are taken by rotating the gantry to 0 and 90°, ensuring that images are obtained from multiple perspectives. These fluoroscopic images are then carefully aligned with DRR (Digitally Reconstructed Radiography) images generated from CT scans of the patients (3–5). This alignment process helps to detect and correct any positioning errors before treatment begins.

One of the key reasons for the widespread adoption of EPID in radiotherapy centers, especially at the grassroots level, is its low operational cost and ease of use. The device is particularly effective at providing clear bony structures images of the patients, making it invaluable for positioning verification in many types of radiotherapy. Its ability to capture real time images quickly has made EPID a practical choice for many facilities, contributing to its sustained popularity.

However, there are certain limitations associated with EPID. Studies have shown that while the device provides sufficiently clear images of bones, the X-ray images it produces can be somewhat blurry, particularly in relation to soft tissue visualization (6). This reduced clarity can make it challenging to accurately distinguish between critical anatomical features such as vertebral bodies and inter vertebral spaces. Although EPID can still identify the contours of organs, its limitations in soft tissue resolution pose challenges for more precise treatments, particularly in areas where tissue contrast is essential. To address these challenges and ensure that radiotherapy is delivered with the highest level of precision, accurate patient positioning is of utmost importance. The success of EPID in providing reliable patient positioning verification largely depends on the skillful alignment of images with DRR, and efforts to maintain high standards of accuracy are crucial for achieving optimal treatment outcomes.

The iSCOUT system, developed domestically, is an independent kilovoltage (kV) X-ray orthogonal image-guidance positioning system, separate from the linear accelerator. It uses kV-level stereoscopic planar imaging technology to capture fluoroscopic images simultaneously from two angles, 135° and 225°, during both positioning and treatment. By aligning these images with reference images based on the patient’s anatomical structures, positioning errors can be identified (7). The kV-level imaging technique primarily uses internal bony structures to correct positioning errors. The iSCOUT system is widely used in various types of tumor radiation therapy, especially in areas that require a high degree of precision, such as head and neck tumors, thoracic tumors, and pelvic tumors. Its multi-angle imaging advantage is particularly effective in pelvic area alignment, as it helps reduce errors caused by organ movement or changes in patient positioning. Studies have shown that rigid structures such as the pelvis and spine are minimally affected by physiological variations and are easily identifiable in kV-level X-ray images, making them ideal for image registration (8, 9).

Additionally, during actual treatments, a single iSCOUT exposure delivers a radiation dose of less than 1 mGy equivalent to only two digital radiography (DR) images-significantly lower than that of EPID. Furthermore, iSCOUTs imaging and registration processes are faster compared to EPID. Since the iSCOUT system operates independently of the accelerator, it does not interfere with the motion mechanisms of the treatment system, ensuring that it does not compromise the precision of the radiotherapy process.

Colorectal cancer (CRC) is a highly prevalent malignancy worldwide, with both incidence and mortality rates steadily increasing in China (10, 11). Studies have shown that preoperative radiotherapy for rectal cancer significantly enhances tumor respectability and increases the likelihood of sphincter preservation, while postoperative radiotherapy greatly reduces recurrence rates (12, 13). Image-guided radiotherapy (IGRT) not only enables precise tumor targeting but also minimizes radiation exposure to surrounding healthy tissues (14, 15). However, in China, advanced IGRT systems are predominantly imported, and many basic radiotherapy facilities lack the financial and technical resources to install such high-end equipment (16, 17). As a result, onboard EPID systems and orthogonal imaging systems have become the mainstream methods for mid-range and lower-tier radiotherapy systems.

This study focuses on evaluating positioning errors in rectal cancer patients undergoing radiotherapy, using the domestically produced iSCOUT image guidance system by Jiangsu Real Company and the onboard EPID system from Varian Medical Systems accelerators. By comparing these two systems, the research aims to provide valuable data for guiding the selection of image- guidance technologies in basic radiotherapy facilities in China. Furthermore, it investigates potential factors that influence positioning errors in rectal cancer radiotherapy, offering insights into optimizing PTV margin expansion.

## 2 Materials and methods

### 2.1 Patient inclusion criteria

A comprehensive retrospective analysis was conducted on rectal cancer patients who underwent treatment with the VARIAN Unique linear accelerator between November 2020 and November 2023. The detailed characteristics of these patients summarized in **Table 1**.

**Table 1:** Baseline characteristics of inclusive patients.

Category	Feature	Percentage
Average Height (m)	1.67 (1.45-1.87)	
Average Weight (kg)	65.7 (37.0-98.8)	
Average BMI (kg/m²)	23.5 (15.4-33.4)	
Age	< 44	15.52%
	45-59	37.55%
	60-74	40.07%
	≥ 75	6.86%
Gender	Male	61.73%
	Female	38.27%
KPS Score	70	1.44%
	80	15.88%
	90	82.68%
Tumor Location	Upper	8.30%
	Upper-Middle and Middle	41.88%
	Lower-Middle and Lower	49.82%
Stage	I	1.81%
	II	6.86%
	III	68.95%
	IV	22.38%
Treatment Type	Preoperative Radiotherapy	51.99%
	Postoperative Radiotherapy	48.01%

The inclusion criteria were as follows: (1) Patients pathologically diagnosed with rectal cancer who received preoperative or postoperative pelvic conventional fractionated radiotherapy; (2) Patients who successfully completed radiotherapy with complete follow-up records and signed informed consent for radiotherapy; (3) Patients who underwent at least six verification sessions during treatment; (4) Patients without other malignant tumors or severe comorbidities; (5) Patients in good general condition with a Karnofsky Performance Status (KPS) score ≥70; (6) Patients positioned in the prone position during localization, with fixation using a “Belly Board”.

Exclusion criteria: (1) Cone-beam computed tomography (CBCT) verification performed fewer than 5 times; (2) Poor compliance with radiotherapy, resulting in failure to complete the full treatment course; (3) Patients with a KPS score of less than 70 or with poor general physical condition; (4) Patients who were unable to comply with the instructions for bladder and rectal preparation.

### 2.2 Positioning and CT simulation

All patients in this study were immobilized using a combination of a belly board (CIVCO, USA) and thermoplastic masks to ensure consistent and reproducible positioning during radiotherapy. The immobilization process began with specific patient instructions: one hour prior to the CT simulation, patients were instructed to completely empty their rectum and drink 1000 ml of water to fully fill the bladder. This preparation is critical, as it helps achieve a consistent internal anatomy by ensuring the bladder is full and the rectum is emptied, both of which are important for optimizing radiation targeting and minimizing dose to nearby healthy tissues (18).

During the simulation, patients’ lower bodies were fully exposed to eliminate any potential errors caused by clothing interference. This allowed for accurate placement and alignment, which is particularly important when treating areas such as the pelvis. Patients were placed in a prone position on the belly board, with the sacrolumbar region positioned at the lower edge of the board’s opening. This strategic positioning allowed the small intestine to drop away from the radiation field, thereby reducing the radiation dose to this sensitive organ. To further enhance stability and minimize motion, patients extended their arms forward and embraced the front of the belly board. Care was taken to avoid any pelvic rotation, as even slight deviations could lead to misalignment and affect treatment accuracy.

Laser guidance positioning lines were drawn directly onto flat areas of the patient’s skin to ensure precise alignment during treatment. A central positioning line was drawn from the treatment center, extending across both sides of the lower back. Additional marks were placed on both iliac joints to serve as reference points for alignment. To ensure further immobilization and accuracy, thermoplastic masks were used to secure the patient’s upper body and prevent any movement. Metal markers were then carefully placed on the masks to indicate the localization center, ensuring precise alignment with the planned treatment area.

Once the patient was securely positioned, CT simulation was conducted with the patient maintaining a full bladder, consistent with earlier preparation instructions. The scan parameters were carefully chosen to provide high-quality images: a tube voltage of 120 kV and current of 150 mAs were used, while the slice thickness and spacing were both set at 5 mm to allow for detailed imaging of the treatment area. The scan covered a large anatomical range, extending from 5 cm above the diaphragm down to the ischial tuberosity. This extensive scanning range ensured that the entire treatment area and surrounding structures were clearly visible, providing the necessary anatomical detail for precise treatment planning and execution. In our center, the CTV-to-PTV margin for patients with rectal cancer is currently defined as a uniform 7 mm expansion in the lateral and anterior–posterior directions, and 10 mm in the superior–inferior (cranio-caudal) direction.

### 2.3 Treatment planning, image guidance system, and registration

After completing the CT simulation, the acquired images were transferred to the Pinnacle planning system (version 9.10). At this stage, the radiation oncologist carefully delineated the target areas, including the tumor and surrounding regions that required treatment, as well as critical organs at risk (OARs) that needed protection from excess radiation exposure. This delineation process was based on the specific characteristics of each patient’s condition, ensuring that the radiation dose would be effectively delivered to the tumor while minimizing damage to healthy tissues. The physician also established prescription doses for the target areas and provided dose constraints for the OARs to guide treatment planning. In addition, all patients were treated using volumetric modulated arc therapy (VMAT) techniques.

Once the physician completed the initial setup, the dosimetrist designed a customized treatment plan based on these parameters. This treatment plan was then submitted for evaluation by a team of specialists to ensure it met all clinical and safety standards. After passing this evaluation, the treatment schedule was finalized. Throughout the first week of radiotherapy, each patient underwent at least three image guidance positioning verifications to ensure that the radiation was delivered to the precise location. In each image-guided verification fraction, both the EPID and iSCOUT image-guidance systems were employed concurrently to acquire setup verification images. The iSCOUT image acquisition system is a sophisticated tool that enhances treatment precision by using two deeply embedded kV X-ray devices. The iSCOUT system features a dual-tube, dual-panel, dual-generator configuration. Its X-ray tubes are positioned at the floor level, while the corresponding flat-panel detectors are suspended from the ceiling. The beam intersection centers of the two tubes coincide with the isocenter of the linear accelerator. The system captures images at non-orthogonal angles 90°, using a 2D-3D bone-matching registration algorithm to verify patient positioning. iSCOUT leverages kV-level stereoscopic planar imaging technology, which is controlled by specialized software. The two X-ray imaging units are symmetrically positioned relative to the localization center, enabling the system to project X-ray images from two directions simultaneously. These images are then used to generate Digital Reconstructed Radiographs (DRRs), which are matched to the patient’s internal anatomy for highly accurate positioning verification.

**Figure 1:**
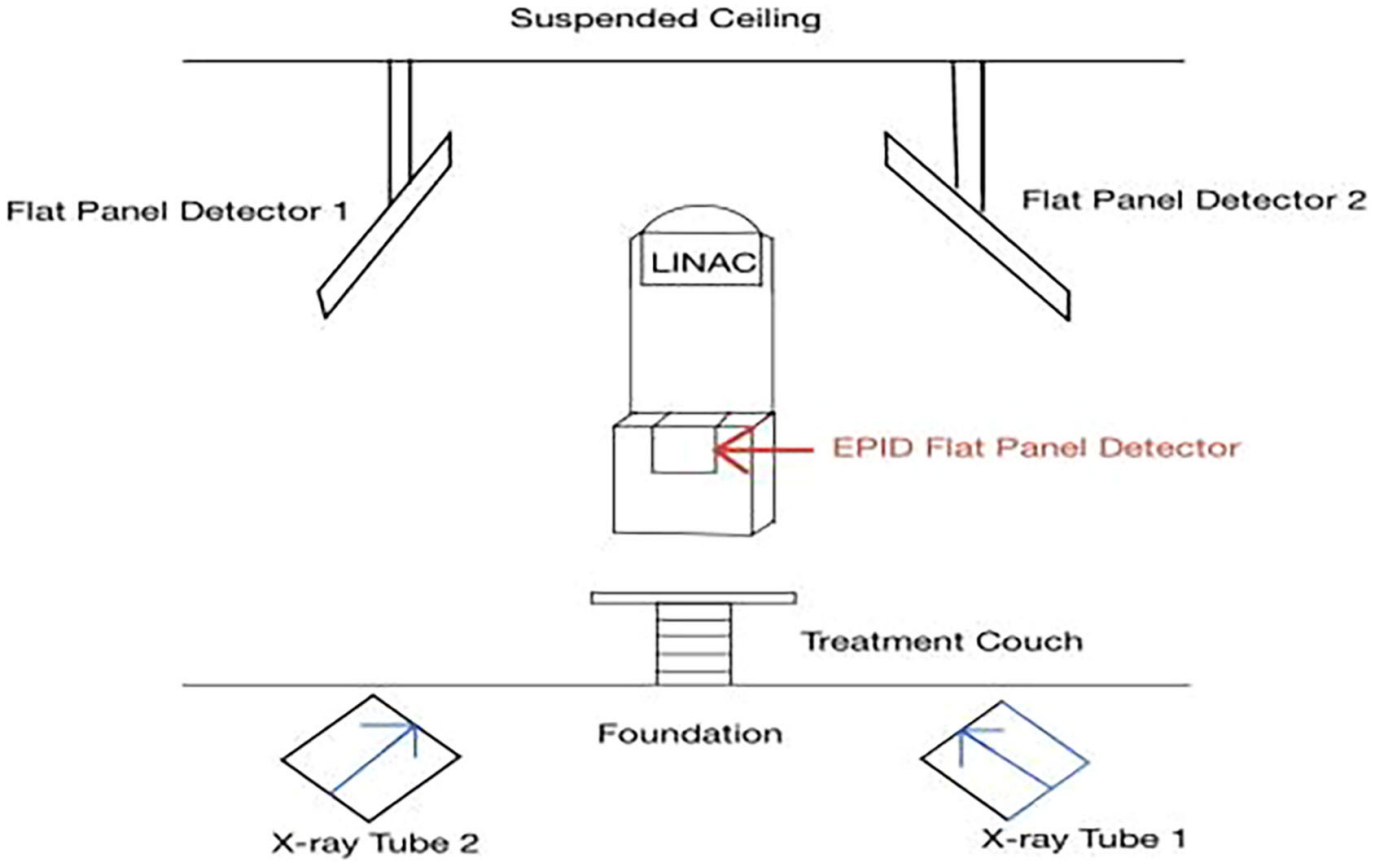
Overall layout of the iSCOUT image-guided system.

**Figure 2:**
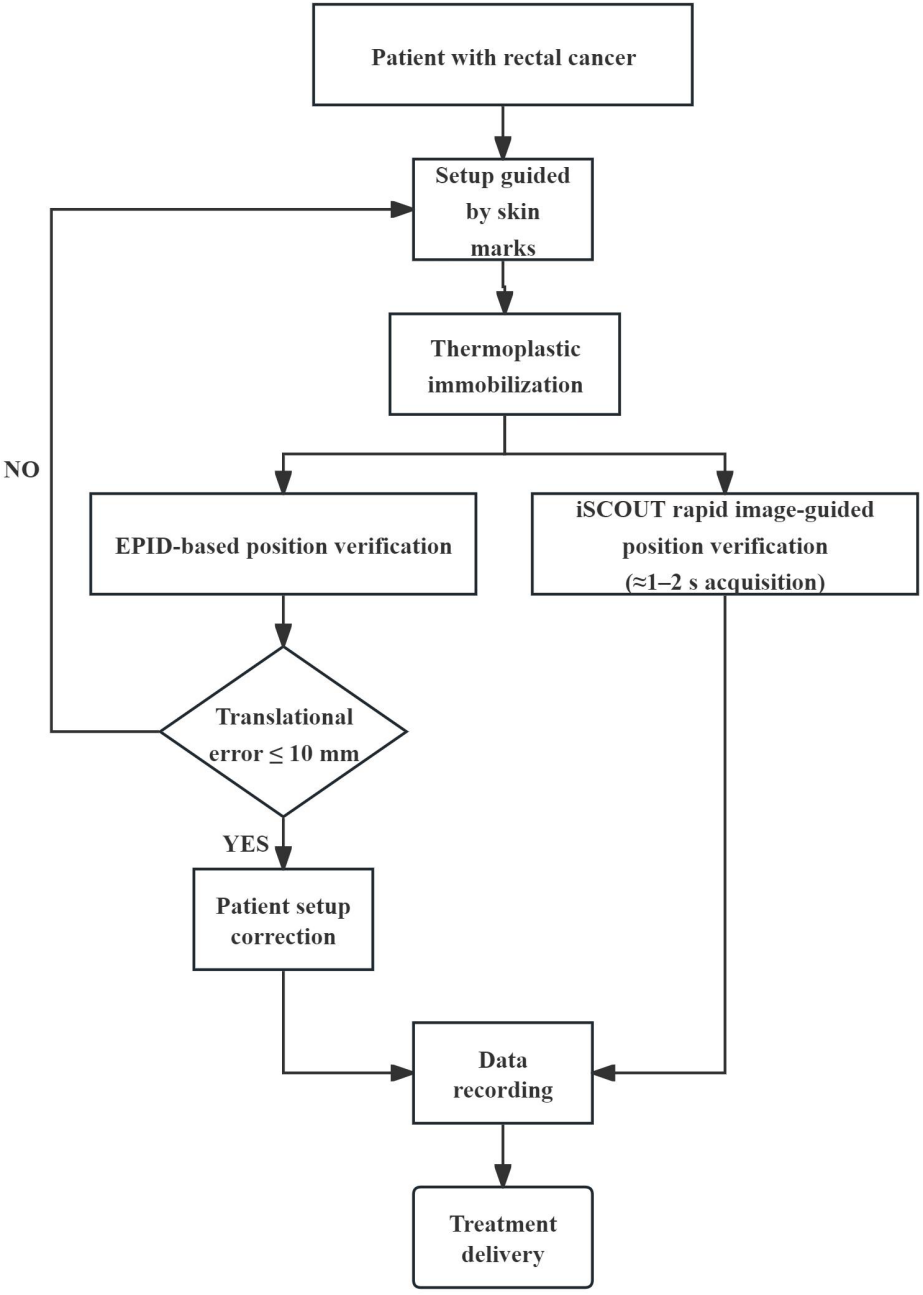
Workflow of the EPID and iSCOUT IGRT systems.

For patients who exhibited significant initial positioning errors during the verification process, the frequency of these checks was increased at the discretion of the supervising physician. According to the protocol of our treatment center, ‘significant initial positioning errors’ are defined as setup deviations exceeding 1 cm in any translational direction. In these cases, additional imaging was conducted to ensure that the patient was properly aligned before each treatment session. Throughout the treatment period, all patients simultaneously underwent EPID and iSCOUT image guidance prior to each fraction of image-guided treatment. The verification process relied on bone registration markers as reference points for precise alignment. Any necessary manual adjustments were made to correct misalignments, and translational errors in the left-right (LR), superior-inferior (SI) and anterior-posterior (AP) directions were recorded for each verification session. Overall layout of the iSCOUT image-guided system and the workflows of the EPID and iSCOUT IGRT systems, as shown in **Figures 1** and **2**.

### 2.4 Calculation of positioning errors and statistical methods

The Positioning errors are categorized into systematic errors and random errors. Systematic error ( Σ) is defined as the standard deviation of the mean error for individual cases, while random error ( σ) is the root mean square (RMS) of the standard deviation of errors for individual cases. The calculation process includes the following steps: (1) Calculate the individual mean positioning error ${\text{m}}_{\text{k}}$ for each case across all treatment sessions, The ${\text{n}}_{\text{k}}$ denotes the number of treatment fractions for the k-th patient, and N is the total number of patients. (2) Determine the individual standard deviation ${\text{S}}_{\text{k}}$ of the positioning errors for each case across all treatment sessions. (3) Compute the mean $\text{M}({\text{m}}_{\text{k}})$ of the individual mean errors across all cases. (4) Calculate the standard deviation of the mean error for each case $\text{Σ }({\text{m}}_{\text{k}})$ using the following formula:


$$\text{Σ }({\text{m}}_{\text{k}})=\sqrt{\frac{\sum {\text{n}}_{\text{k}}{\left({\text{m}}_{\text{k}}-\text{M}({\text{m}}_{\text{k}})\right)}^{2}}{\text{N}-1}\;}$$

5. Obtain the standard deviation of the individual case errors $\text{σ}({\text{S}}_{\text{k}})$ using the following formula:


$$\text{σ}({\text{S}}_{\text{k}})=\sqrt{\frac{\sum {\left({\text{S}}_{\text{k}}\right)}^{2}}{\text{N}-1}}$$

To ensure that 90% of patients receive the prescribed dose in the clinical target volume (CTV) at 95% or above, the PTV margins (MPTV) in the LR, SI, and AP directions were calculated by the following equation (19):


MPTV=2.5 Σ+0.7σ

### 2.5 Statistical analysis

Data analysis was conducted using SPSS 25.0 statistical software, ensuring that the statistical methods applied were robust and appropriate for the study design. For the analysis of quantitative data, the results were expressed as ( Σ±σ), where Σ represents the standard deviation of the individual mean errors, indicating the systematic error, and σ denotes the root mean square (RMS) of the standard deviation of the individual errors, reflecting the random error. This dual representation of errors helped differentiate between consistent deviations in patient positioning (systematic error) and random fluctuations that occurred across different radiotherapy sessions (random error). To compare the registration results obtained from these two image-guidance systems, paired-sample t-tests were employed.

This statistical method was chosen because it allows for the comparison of two sets of related data, making it ideal for analyzing the differences in displacement errors measured by different registration methods (e.g., EPID vs. iSCOUT) for the same patients during radiotherapy. The mean displacement errors across all directions (LR, SI, and AP axes) were then further analyzed using Pearson correlation for continuous variables, providing insights into any potential relationships between these variables and other clinical factors, such as body mass index (BMI), age, or tumor location. The analysis of BMI distribution for systematic error and kappa consistency was conducted by categorizing patients into four groups based on BMI: underweight (BMI < 18.5), normal weight (18.5 ≤ BMI ≤ 24), overweight (24.0 ≤ BMI ≤ 28), and obese (BMI ≥ 28) (20). In 2022, the United Nations World Health Organization (WHO) conducted an assessment of global human physical fitness and life expectancy, resulting in updated age classification standards. According to these new guidelines, individuals under the age of 44 are considered young adults, those between 45 and 59 are categorized as middle-aged, those age in the range of 60 to 74 are classified as young elderly, and the patients above 75 years old are considered elderly. The rectal tumors location were classified according to their anatomical location relative to the dentate line: lesions located within 5 cm from the dentate line were defined as lower rectal cancer, those between 5 and 10 cm as middle rectal cancer, and those between 10 and 15 cm as upper rectal cancer.

In addition, independent-sample t-tests were applied to compare displacement errors between two groups, such as male vs. female patients or different age cohorts. This test determined whether there were statistically significant differences in the mean positioning errors between groups. For comparisons involving multiple groups, such as patients grouped by BMI categories or age brackets, multiple t-tests were employed to assess the variation in means across these groups. This step ensured that all relevant comparisons were made, highlighting any significant differences in positioning accuracy across patient demographics or clinical characteristics. Kappa analysis was performed to assess the agreement between EPID and iSCOUT in measuring patient setup errors. The Kappa test is a statistical method used to evaluate the level of agreement among two or more methods in classifying the same set of subjects, particularly suitable for nominal or ordinal categorical data. A higher k value indicates better agreement. In this study, setup errors were classified into three categories based on clinically acceptable thresholds: ≤ 2 mm, > 2 mm and ≤ 4 mm, and > 4 mm. The continuous setup deviations measured by the two image verification systems were categorized accordingly, and the consistency of setup error classification between the iSCOUT and EPID systems was evaluated using the Kappa statistical test. For the consistency levels of the kappa statistic, the k values are interpreted as follows: 0 = poor, 0.01 - 0.20 = slight, 0.21 - 0.40 = fair, 0.41 - 0.60 = moderate, 0.61 - 0.80 = substantial, and 0.81 - 1.00 = almost perfect agreement (21). Throughout the data analysis, a P-value of less than 0.05 was considered statistically significant.

## 3 Results

### 3.1 Comparison of the positioning error

**Figure 3:**
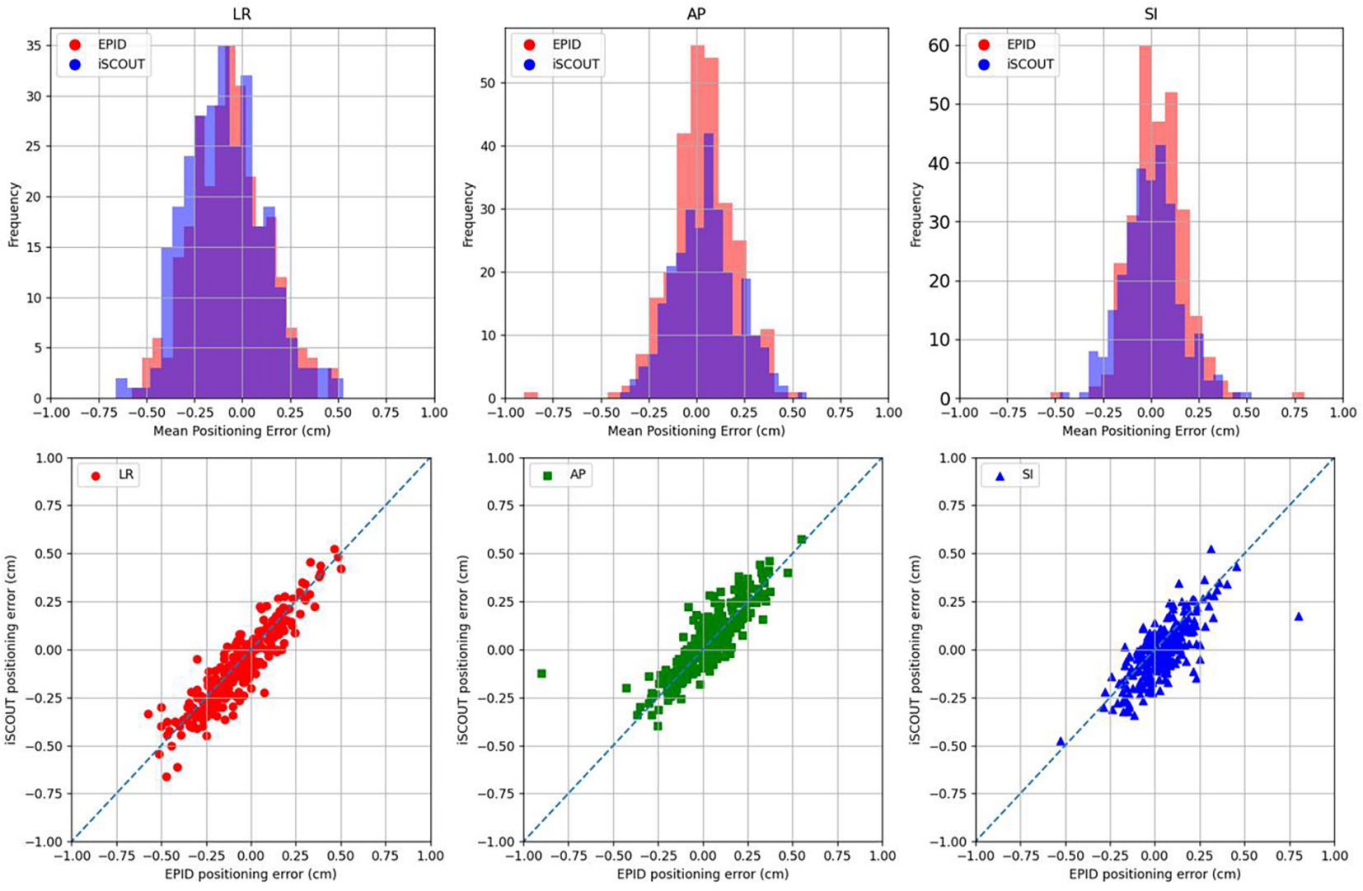
The positioning error distribution for LR, SI, AP directions (Upper row) and the correlation between iSCOUT and EPID for all inclusive patients (Lower row).

The upper part of **Figure 3** shows the average positioning data in the LR, SI, and AP directions for 277 patients. It can be observed from the figure that the positioning data in all three directions follow a Gaussian-like distribution. The lower part compares the average positioning data for all patients as measured by EPID and iSCOUT. Since all measured positioning error values that fell within the clinical tolerance (i.e., three-dimensional translation errors were all less than 1.0 cm) were included in the final analysis to accurately reflect the complete picture of clinical practice, although a notable outlier (LR, AP, SI: 0.8, -0.3, -0.9 cm) was observed. This outlier datum was confirmed to be a valid clinical measurement and was therefore retained. As shown in **Table 2**, the systematic and random errors of the enrolled patients, as well as the kappa consistency analysis between the two image-guidance systems are presented in **Table 2**. In both the SI and AP directions, the differences were statistically significant (P < 0.05). Across all three directions, the kappa values were greater than 0.5 (k = 0.794, 0.852, 0.610). Notably, the kappa values for the LR and SI directions in both the iSCOUT and EPID groups were above 0.7, indicating a high level of consistency.

**Table 2:** Comparison of setup errors ( Σ± σ, cm) between EPID and iSCOUT.

Direction	EPID	iSCOUT	p	k
LR	0.16 ± 0.23	0.15 ± 0.25	0.189	0.794
SI	0.19 ± 0.31	0.19 ± 0.31	0.000	0.852
AP	0.13 ± 0.18	0.14 ± 0.19	0.000	0.610

### 3.2 Tumor location

**Figure 4:**
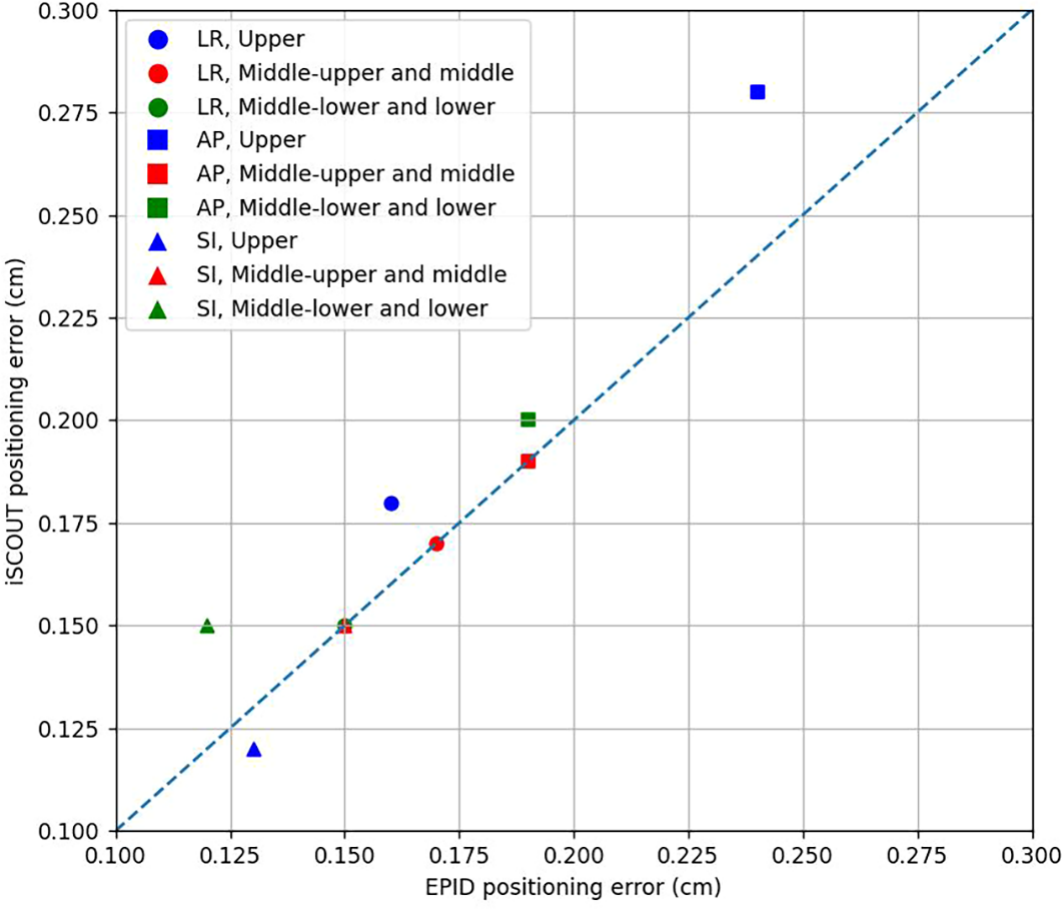
The mean positioning error of iSCOUT and EPID for different rectal tumor locations.

The analysis results of positioning errors between the iSCOUT and EPID systems at different rectal cancer locations in **Figure 4** and **Table 3**. Significant differences in setup errors were observed among the patient groups in the SI and AP directions. In the LR and SI directions, the kappa values for error data across groups were all greater than 0.78, indicating a high level of agreement, whereas in the AP direction, the kappa values for patient error data were lower than those in the LR and SI directions. Overall, the two systems perform consistently well in the LR and SI directions, while the AP direction presents greater variability and lower consistency. **Table 4** indicates the PTV margin expansion boundaries calculated based on the positioning error results from the two image-guidance systems for various primary locations of rectal cancer. Overall, the PTV expansion in the SI direction is larger for tumors located in the upper rectum compared to those in the middle and lower locations.

**Table 3:** Comparison of p-values and k-values between upper rectum and middle/upper-middle rectum locations.

Direction	Upper		Mid & upper-mid		Lower & lower-mid	
	p	k	p	k	p	k
LR	0.147	0.817	0.415	0.780	0.653	0.801
SI	0.004	0.916	0.000	0.837	0.000	0.847
AP	0.027	0.629	0.000	0.637	0.000	0.584

**Table 4:** PTV expansion (cm) calculation results for different rectal tumor locations of EPID and iSCOUT.

Direction	Upper		Mid & upper-mid		Lower & lower-mid	
	EPID	iSCOUT	EPID	iSCOUT	EPID	iSCOUT
LR	0.57	0.63	0.59	0.59	0.54	0.55
SI	0.85	0.96	0.69	0.69	0.69	0.72
AP	0.44	0.43	0.51	0.52	0.42	0.5

### 3.3 BMI

**Figure 5:**
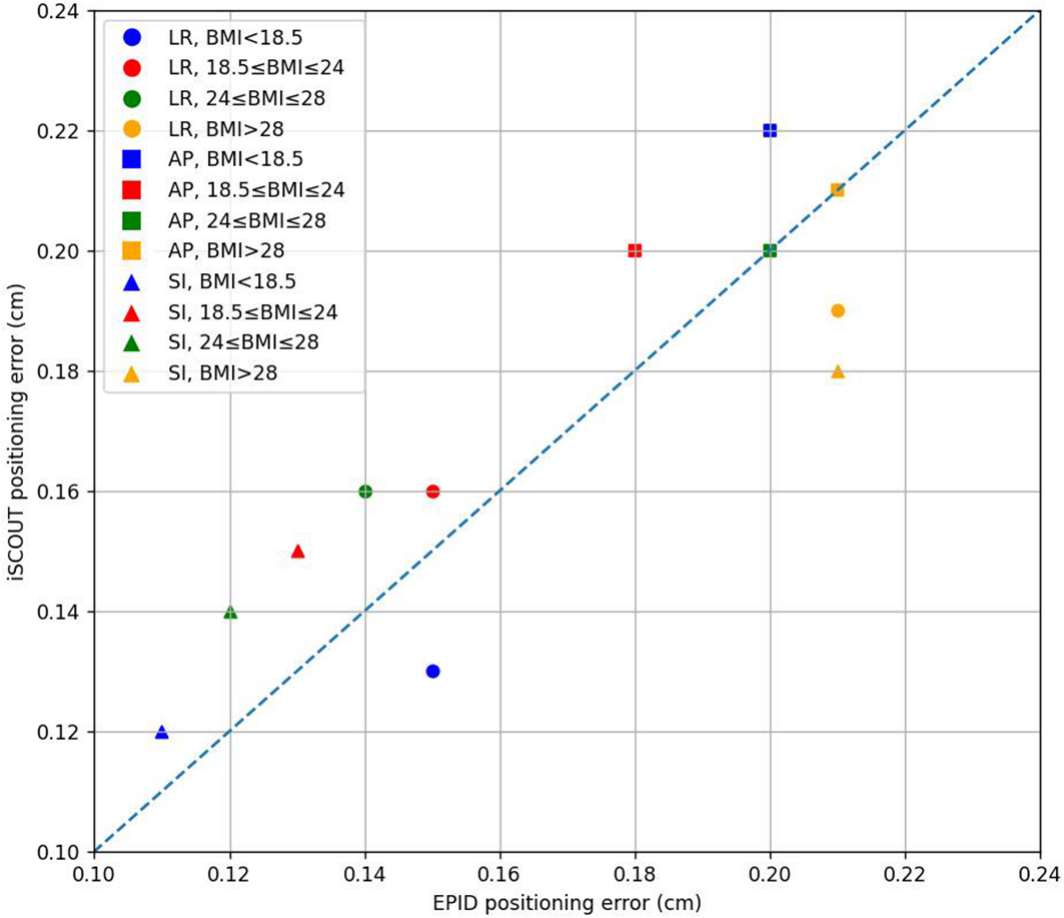
The correlations of positioning errors in 3 directions between iSCOUT and EPID systems for different BMI groups.

From the positioning error results of the EPID and iSCOUT systems for different BMI groups in **Figure 5** and **Table 5**, the results show that in the LR direction, there are no significant differences between the two systems, and the consistency remains high with Kappa values above 0.76 across all BMI categories. In the SI direction, significant differences in positioning errors are observed in the normal weight (18.5≤BMI ≤ 24) and overweight (24.0≤BMI ≤ 28) groups, but both systems demonstrate strong consistency with Kappa values exceeding 0.84. It can be observed that for the underweight group, the consistency between the two systems in the AP direction was relatively low. However, as BMI increases, the consistency in the AP direction improves. For obese patients, the PTV margin expansion was larger compared to other groups, indicating a need for greater caution in planning to account for positioning errors in this population (**Table 6**).

**Table 5:** p and k values for different BMI groups.

Direction	BMI < 18.5		18.5≤ BMI ≤ 24		24.0< BMI < 28		BMI ≥ 28	
	p	k	p	k	p	k	p	k
LR	0.558	0.866	0.341	0.807	0.464	0.762	0.305	0.761
SI	0.780	0.765	0.000	0.846	0.000	0.874	0.480	0.813
AP	0.003	0.409	0.000	0.578	0.000	0.650	0.077	0.694

**Table 6:** PTV expansion (cm) of the two systems for different BMI groups, all units are in centimeter.

Direction	BMI < 18.5		18.5≤ BMI ≤ 24		24.0< BMI < 28		BMI ≥ 28	
	EPID	iSCOUT	EPID	iSCOUT	EPID	iSCOUT	EPID	iSCOUT
LR	0.55	0.50	0.55	0.58	0.51	0.55	0.66	0.62
SI	0.70	0.71	0.67	0.71	0.71	0.73	0.76	0.78
AP	0.40	0.43	0.44	0.50	0.44	0.49	0.65	0.59

### 3.4 Age

**Figure 6:**
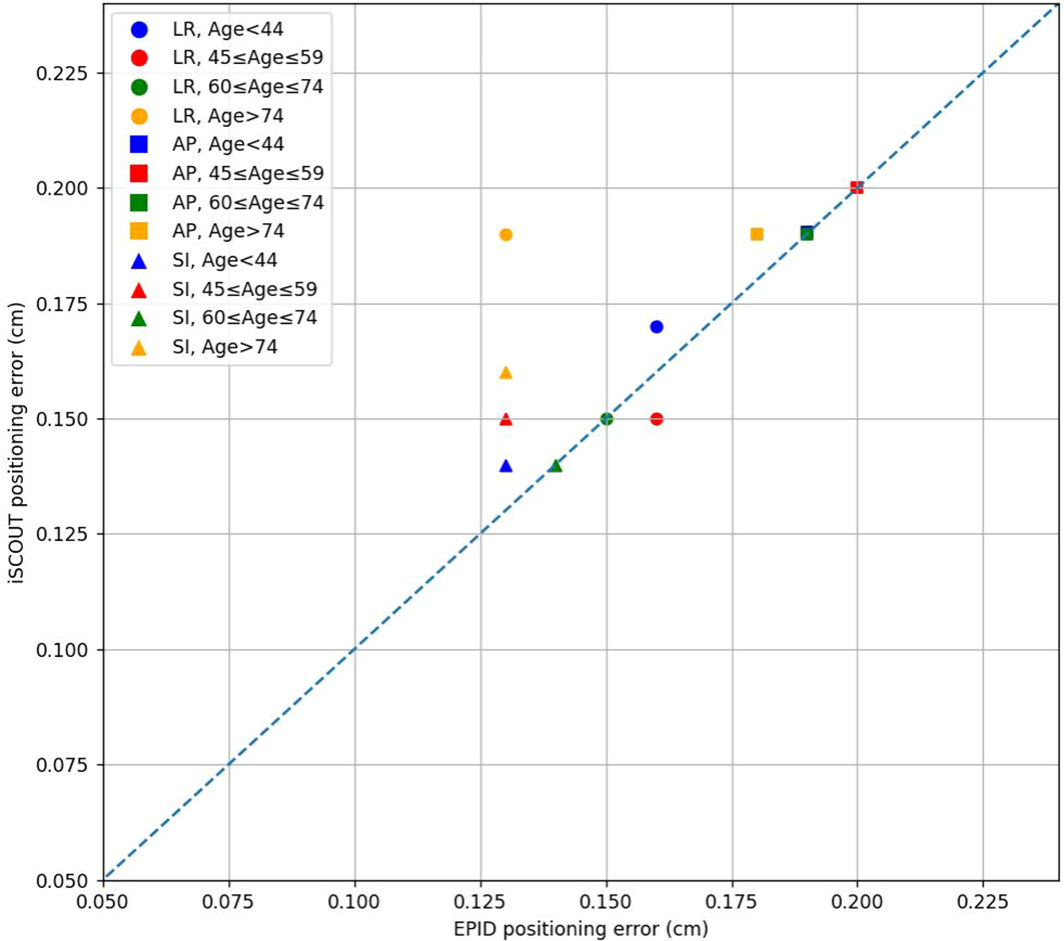
The correlations of positioning errors in 3 directions between iSCOUT and EPID systems for different age groups.

The analysis of positioning errors between the EPID and iSCOUT systems across different age groups in **Figure 6** and **Table 7** shows that in the LR direction, there are no significant differences between the two systems for patients under 44 years and those aged 60 to 74 years. However, for the 45 to 59 years group, there is a significant difference (p=0.002) with strong consistency (Kappa = 0.826), while the 75 to 89 years group shows moderate consistency (Kappa = 0.777). In the SI direction, the systems show no significant differences in positioning errors for the 44 years and below group, but in other age groups, significant differences are present (p < 0.05). Despite this, the consistency remains high, with Kappa values ranging from 0.836 to 0.868. For the AP direction, there are significant differences in the 45 to 59 years and 60 to 74 years groups, but the consistency is relatively lower, particularly for the 75 to 89 years group, where the Kappa value is 0.570, indicating moderate agreement. **Table 8** presents the calculated PTV margin expansion results for different age groups. Across all age groups, the PTV margin expansion in the SI direction was larger than that in the LR and AP directions.

**Table 7:** p and k-values for different age groups.

Direction	<44 years		45–59 years		60–74 years		75–89 years	
	p	k	p	k	p	k	p	k
LR	0.598	0.827	0.002	0.826	0.134	0.752	0.082	0.777
SI	0.204	0.931	0.003	0.836	0.000	0.868	0.009	0.868
AP	0.396	0.615	0.000	0.593	0.000	0.633	0.137	0.570

**Table 8:** PTV expansion (cm) of the two systems for different age groups.

Direction	<44 years		45–59 years		60–74 years		75–89 years	
	EPID	iSCOUT	EPID	iSCOUT	EPID	iSCOUT	EPID	iSCOUT
LR	0.60	0.61	0.56	0.55	0.54	0.54	0.49	0.55
SI	0.71	0.69	0.72	0.72	0.69	0.69	0.70	0.72
AP	0.49	0.50	0.45	0.51	0.48	0.48	0.45	0.53

### 3.5 Gender

**Figure 7:**
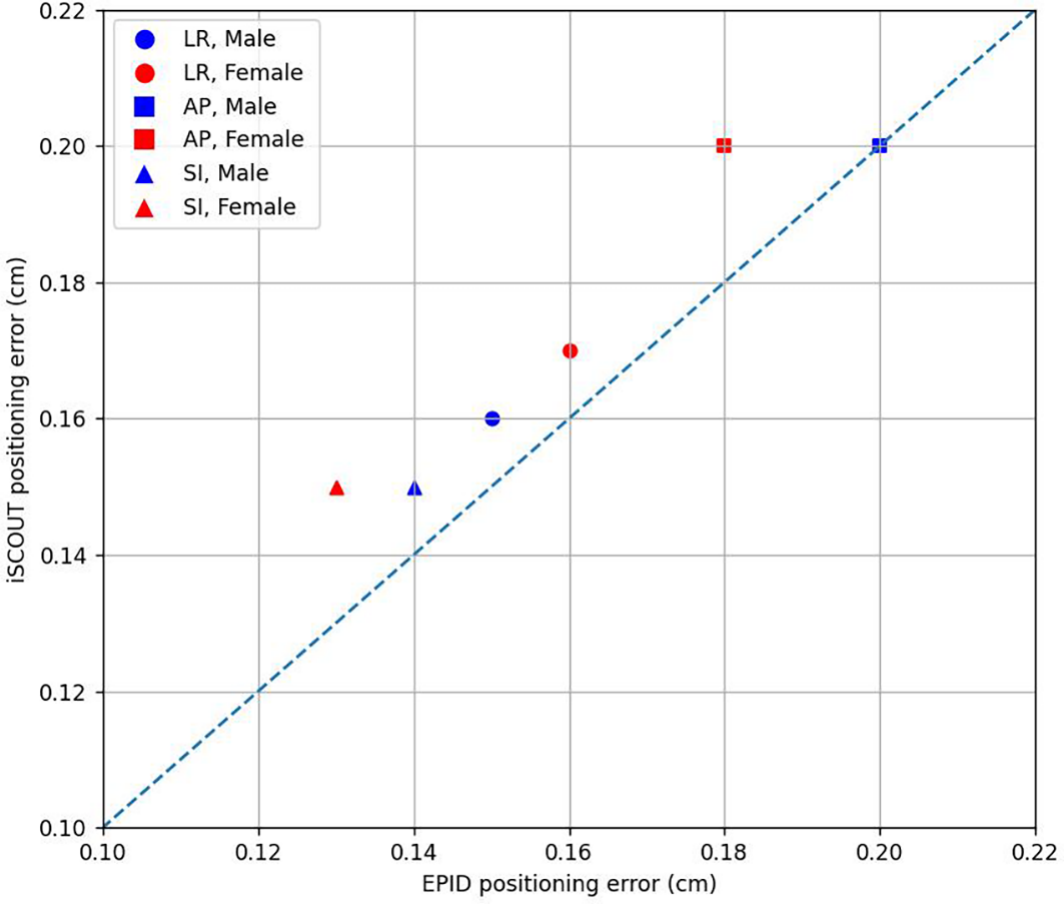
The correlations of positioning errors in 3 directions between iSCOUT and EPID systems for different gender groups.

**Figure 7** and **Table 9** present the data regarding positioning errors based on gender. For the LR direction, both EPID and iSCOUT show no significant differences in positioning errors between males and females (p > 0.05). In the SI direction, there are statistically significant differences for both systems, with values for males being slightly higher than those for females. Similarly, in the AP direction, significant differences are also observed, with males showing higher errors compared to females. The kappa values indicate good consistency for both genders across all directions, particularly in the SI direction where both systems exhibit high agreement.

**Table 9:** p and k values for male and female groups.

Direction	Male		Female	
	p	k	p	k
LR	0.081	0.797	0.959	0.790
SI	0.000	0.852	0.000	0.851
AP	0.000	0.639	0.000	0.567

**Table 10** present the analysis results of positioning errors and PTV margin expansion based on gender grouping. From a gender perspective, there were no observed differences in positioning errors or margin expansion results. However, the results obtained from the iSCOUT system were generally higher than those from the EPID system. In terms of consistency between the two systems, the consistency in the AP direction was slightly lower compared to that in the LR and SI directions. Similarly, the PTV margin expansion results did not show any notable variation based on gender, indicating that gender does not have a significant effect on positioning accuracy or the expansion of the PTV margins.

**Table 10:** PTV expansion (cm) of the two systems for different gender groups.

Direction	Male		Female	
	EPID	iSCOUT	EPID	iSCOUT
LR	0.54	0.57	0.56	0.60
SI	0.71	0.72	0.67	0.72
AP	0.48	0.51	0.45	0.51

## 4 Discussion

Image-guidance registration technology has been continuously developed as an important means to improve the precision of radiotherapy and ensure the quality of treatment positioning in recent years (22, 23). It plays an increasingly vital role in the radiotherapy of various cancer patients (24–27).

As shown in **Table 2**, positioning errors in the SI and AP directions exhibited statistical significance (P < 0.05) when using EPID and iSCOUT for patient registration. The error values from both groups were quite close, with Kappa values in the LR and AP directions exceeding 0.6, indicating high consistency; in the SI direction, the Kappa value exceeded 0.8, demonstrating very high consistency. This high level of agreement may be attributed to the near-simultaneous image acquisition of both systems within the same pre-treatment session and the extremely short exposure time, which minimizes the impact of intra-fractional motion and organ positional variation. Furthermore, both the EPID and iSCOUT systems had good recognition of the pelvis, leading to higher registration accuracy. The use of a belly board effectively controlled the movement of the small intestine, reducing radiation exposure to critical organs, thus ensuring stability and reproducibility during treatment. In summary, equipping the accelerator with the iSCOUT system, in conjunction with the EPID for registration, can significantly enhance registration efficiency. In this study, patients were categorized based on tumor location into three groups: upper rectum, middle and upper rectum, and lower rectum and middle lower rectum. The study found that positioning errors during registration with EPID and iSCOUT were very similar across all three directions, with statistical significance observed in the SI and AP directions. The Kappa values for positioning errors in the LR and SI directions were approximately 0.8 or higher, indicating high consistency. However, the Kappa value in the AP direction was only around 0.6, suggesting lower consistency compared to the other two directions. This may be due to the fact that the registration in the AP direction for EPID only requires reference from the anterior X-ray image of the patient, aligning according to deviations of the spine and pelvis, which may also be related to the bladder filling and urine volume prior to treatment. In contrast, the iSCOUT X-ray images were obtained from two angles of 135° and 225°. The movement of images during registration directly affected the positioning in the LR and AP directions. Additionally, since the images were captured at a 45° inclination, any rotation of the patient’s body during positioning would significantly affect the registration with iSCOUT.

Regarding the PTV margin expansion for the three patient groups, the expansion range in the LR and AP directions was generally similar. However, in the SI direction, the PTV margin for the upper rectum group was significantly larger than for the other two groups. This result may be attributed to the anatomical location of upper rectal tumors, which are situated approximately 10–15 cm from the anal margin, higher in position. During image registration, alignment may rely more on stable bony landmarks adjacent to the upper rectum, such as the vertebral bodies. In contrast, the middle and lower rectal tumors are positioned deeper within the pelvis, typically aligning with landmarks such as the femoral heads, pubic symphysis, and sacrum, which may be more susceptible to pelvic motion and positional variability. Additionally, the number of patients in the upper rectum group was significantly smaller than in the other two groups, which may also affect the results.

Patients were categorized into four BMI groups based on their BMI values: BMI < 18.5 for underweight, 18.5 ≤ BMI ≤ 24 for normal weight, 24.0 ≤ BMI ≤ 28 for overweight, and BMI ≥ 28 for obesity. From the positioning error results of the EPID and iSCOUT systems shown in **Table 5**, it can be concluded that the consistency in the AP direction was lower for the underweight group. As body weight increased, the consistency in the AP direction gradually improved for both systems. In the LR and SI directions, both systems exhibited high consistency. The overall PTV margin expansion across the four groups followed an increasing trend from BMI < 18.5 to BMI ≥ 28, with differences observed in all three directions.

Based on age, patients were divided into four groups: those younger than 44 years (youth group), 45 to 59 years (middle-aged group), 60 to 74 years (older adult group), and those 75 years and older (elderly group). Among the four groups, the positioning errors in the LR, SI, and AP directions for the middle-aged group showed no significant differences, and statistical significance was noted in the older adult group for the SI and AP directions. The elderly group exhibited statistical significance in the SI direction, while no significance was observed in other directions. The Kappa values for the SI direction in all four groups were greater than 0.8, indicating good consistency. For the LR direction, the Kappa values for the youth and middle-aged groups exceeded 0.8, showing good consistency, while those for the older adult and middle-aged groups ranged between 0.7 and 0.8, indicating moderate consistency. In the AP direction, the Kappa values for the youth and older adult groups exceeded 0.6, indicating moderate consistency, while the Kappa values for the middle-aged and elderly groups were less than 0.6, indicating lower consistency. In **Table 8**, the PTV margin in the AP direction for the middle-aged and elderly groups was larger post-registration with iSCOUT compared to EPID, with a similar trend observed in the LR direction for the elderly group. This may be due to older patients potentially exhibiting unintentional body rotation during positioning, resulting in poorer consistency in the LR and AP directions compared to younger patients. Additionally, the smaller sample size in the elderly group could also impact the results. For patients of different genders, the positioning errors in the LR, SI, and AP directions showed little difference between male and female patients, with Kappa values remaining consistent and significant in the SI and AP directions. The PTV margins for both groups were also generally consistent, indicating that gender has minimal impact on the registration results of EPID and iSCOUT.

For patients with significantly deviating data points, the author observed in clinical practice that discrepancies in the positioning images for rectal cancer patients may arise from variations in bladder filling, differences in the angles of the two systems when capturing images, and rotation errors during bone registration. In conclusion, the tumor location and patient BMI had the most substantial impact on the consistency of the registration results of EPID and iSCOUT, followed by age, with gender having the least impact. Additionally, the tumor location and age can influence patient positioning errors, consequently affecting the PTV margin. Overall, the PTV margin expansion followed an increasing trend from BMI < 18.5 to BMI ≥ 28. Therefore, in clinical practice, physicians should consider patients’ age and tumor location to make appropriate decisions regarding PTV expansion.

This study does have some limitations: Firstly, it is a single center, retrospective study. Given the radiation exposure of iSCOUT, using different fixation devices for the same patient during positioning could lead to unnecessary exposure, which raises ethical concerns. Future studies with larger sample sizes would help balance inter group factors, minimizing biases inherent in retrospective studies while avoiding ethical issues associated with prospective controlled studies. Secondly, the review of data by treatment therapists at the center may result in some false-positive results. To address this limitation, the verification images were uploaded to the system for review by the supervising physician on the same day, with timely feedback provided to minimize related impacts. Thirdly, this study focused solely on rectal cancer patients fixed using the belly board, with a single sample size and a significantly lower number of upper rectum patients, potentially affecting the study results. Future research could involve additional treatment sites and fixation devices to enhance clinical studies. Fourthly, while CBCT is considered the gold standard for image registration due to its ability to accurately reflect the relationship between the target area and surrounding critical organs, this study only employed EPID and iSCOUT for registration. Both methods have poorer imaging quality for soft tissues, with registration based on bony landmarks and no CBCT validation conducted. Future prospective cohort studies should further compare the advantages and disadvantages of various imaging verification systems. In analysing positioning errors for rectal cancer patients during radiotherapy based on tumor location, age, and gender, no significant differences were observed. Therefore, in cases of EPID system malfunction or other situations, the iSCOUT image-guidance positioning system can serve as an alternative to the EPID imaging positioning system. However, at present, the X-ray images acquired by the iSCOUT system cannot be transmitted back to the hospital information system, which limits the convenience for clinicians to review images remotely. This represents a practical limitation in routine clinical use. We acknowledge that addressing this issue in the future would further enhance the clinical applicability of iSCOUT. Furthermore, during rectal cancer treatment, variations in PTV margin due to different pathological locations are present, following a specific pattern: as the rectal tumor location moves upward from the lower location, the positioning error in rectal cancer patients tends to increase. When treating patients in the prone position with a belly board for personalized treatment, the PTV for the lower, middle, and upper rectum should be expanded separately.

## Data Availability

The raw data supporting the conclusions of this article will be made available by the authors, without undue reservation.
